# Construction of integrative transcriptome to boost systematic exploration of *Bougainvillea*

**DOI:** 10.1038/s41598-022-04984-8

**Published:** 2022-01-18

**Authors:** Qi Luo, Ziliang Chen, Tingting Xu, Dangzheng Huang, Haitao Hou, Chenjie Hong, Fulin Zhan, Hangqi Guo, Zhe Lin, Xiaoling Guo, Liang Chen, Zhi-Liang Ji

**Affiliations:** 1 Xiamen Highway Development Center, Xiamen, 361008 Fujian Province China; 2 The Wanyin Environmental Technology Limited Company, Xiamen, 361006 China; 3 School of Life Sciences, Xiamen University, Xiamen, 361102 China; 4 College of the Environment and Ecology, Xiamen University, Xiamen, 361102 China

**Keywords:** Plant biotechnology, Bioinformatics

## Abstract

Members of the genus *Bougainvillea* are rich sources of natural dyes, pigments, and traditional medicines. They are also commonly used as ornamentals in roadside landscape construction. However, the horticultural development of *Bougainvillea* flowers with extended growth periods and coloration is not always feasible. One reason is limited molecular knowledge and no genomic information for *Bougainvillea*. Here, we compiled an integrative transcriptome of all expressed transcripts for *Bougainvillea* × *buttiana* Miss Manila by integrating 20 Illumina-sequencing RNA transcriptomes. The integrative transcriptome consisted of 97,623 distinct transcripts. Of these, 47,006 were protein-coding, 31,109 were non-coding, and 19,508 were unannotated. In addition, we affirmed that the integrative transcriptome could serve as a surrogate reference to the genome in aiding accurate transcriptome assembly. For convenience, we curated the integrative transcriptome database for *Bougainvillea*, namely InTransBo, which can be freely accessed at http://www.bio-add.org/InTransBo/index.jsp. To the best of our knowledge, the present study is the most comprehensive genomic resource for *Bougainvillea* up-to-date. The integrative transcriptome helps fill the genomic gap and elucidate the transcriptional nature of *Bougainvillea*. It may also advance progress in the precise regulation of flowering in horticulture. The same strategy can be readily applied toward the systematic exploration of other plant species lacking complete genomic information.

## Introduction

*Bougainvillea* sp. are native to the Amazonian rainforests of South America but are globally distributed. They are used mainly as ornamental and landscaping plants^1^. *Bougainvillea* sp. belongs to the family Nyctaginaceae and comprises ~ 18 species (*B. berberidifolia, B. buttiana, B. campanulata, B. glabra, B. herzogiana, B. infesta, B. lehmanniana, B*. *lehmannii, B. malmeana, B. modesta, B. pachyphylla, B. peruviana, B. pomacea, B. precox, B. spectabilis, B. spinosa, B. stipitata,* and *B. trollii*)^2^. To date, over 100 cultivars and three major hybrids have been recognized^1,2^. However, only four species (*B. buttiana, B. glabra, B. spectabilis,* and *B. peruviana*) have been commercially exploited^3^.

*Bougainvillea* sp. have been extensively investigated since 1970 as potential sources of traditional medicine^4,5^. Aqueous extracts and decoctions of *Bougainvillea* were used for fertility control by the tribal people of several countries^6^. *Bougainvillea* may also have anticancer, antidiabetic, antihepatotoxic, anti-inflammatory, antihyperlipidemic, antimicrobial, antioxidant, and anti-ulcer properties^7^. The alkaloids, essential oils, flavonoids, glycosides, oxalates, phenolics, phlobotannins, quinones, saponins, tannins, and terpenoids in *Bougainvillea* sp. might account for their putative medicinal properties^8^. Bougainvinones, pinitol, quercetagetin, quercetin, and terpinolene may also contribute to the therapeutic efficacy of *Bougainvillea*^4,6^.

Analyses of the metabolites^9^, natural dyes and pigments^9^, medicinal uses, and species diversity of *Bougainvillea* have been conducted. In contrast, there have been few molecular studies of this genus. No genome of *Bougainvillea* has ever been sequenced up-to-date; and it won’t be done in the coming few years due to technical and economic difficulties. Limited omics research has been performed to elucidate the molecular basis of the aforementioned properties of *Bougainvillea* especially at the systematic level. As no genome has been clarified for *Bougainvillea,* current molecular research on this plant is often compared against or referred to *Arabidopsis thaliana*. Consequently, its gene behavior is uncertain, ambiguous, or even misunderstood as there are genomic gaps between organisms. Therefore, an alternative genomic resource is required that can complement or fill the no-genome gap. Here, our objectives were to use multiple Illumina RNA sequencing (RNA-seq) transcriptomes determined for various *Bougainvillea* tissues and generate an integrative transcriptome, which consisted of all expressed transcripts. This integrative transcriptome could serve as an alternative genomic reference for the molecular exploration of *Bougainvillea* and will be presented as an online interactive database.

## Materials and methods

### Sample collection

The tissue samples were collected from the pot-culture *Bougainvillea* (*Bougainvillea* × *buttiana* Miss Manila) at the same time point of normal flower period (from September to November) at Xiamen city, PR China. The samples comprised of thorns, buds, bracts, leaves, stems, and flowers (Table 1). The tissues were excised either from different parts of the same plant or from different plants at the same time point, washed with distilled water, and briefly air-dried in a clean environment. The tissues were mixed, randomly divided into two replicates, packed in silver paper, frozen in liquid nitrogen, and stored in the School of Life Science, Xiamen University, Chen’s lab.

**Table 1:** Illumina RNA-sequencing datasets used in this study.

Tissue/dataset	Raw reads	Clean reads	Raw base (G)	Clean base (G)	Q30 (%)	GC content (%)
Small leaf	33,613,973	31,358,904	10.08	9.41	93.60	43.13
Stem under bud	28,729,693	27,644,873	8.62	8.29	93.09	43.16
Stem under flower	26,343,968	25,698,944	7.90	7.71	90.50	42.95
Thorn (lateral)	34,229,176	32,719,720	10.27	9.82	93.73	42.91
Bud (lateral)	31,318,836	23,553,700	9.39	7.07	93.24	42.84
Bud of about to bloom (lateral)	29,759,552	21,379,446	9.93	8.63	85.86	42.55
Bracteole of just blooming (lateral)	31,247,574	30,110,664	9.37	9.03	93.90	42.92
Bract of half open blossom (lateral)	32,190,609	30,967,796	9.66	9.29	93.62	42.38
Bract of fully open blossom (lateral)	30,698,035	30,294,725	9.21	9.09	93.72	42.41
Leaf sprout (top)	29,239,014	28,026,332	8.77	8.41	93.62	42.80
Bud (top)	32,856,663	31,772,572	9.86	9.53	92.94	42.85
Bud of about to bloom (top)	33,433,464	32,960,179	10.03	9.89	93.65	42.87
Bracteole of just blooming (top)	30,645,057	30,328,864	9.19	9.10	90.81	42.80
Bract of half open blossom (top)	29,613,243	29,302,625	8.88	8.79	93.74	42.50
Bract of fully open blossom (top)	30,698,035	30,294,725	9.21	9.09	93.72	42.41
Flower of just appear	33,460,411	31,701,317	10.04	9.51	94.24	42.99
Flower of about to bloom	30,982,149	29,649,261	9.29	8.89	90.09	42.89
Flower of just blooming	33,115,209	31,568,381	9.93	9.47	90.82	42.69
Flower of half open blossom	32,141,549	30,546,299	9.64	9.16	93.45	42.90
Flower of fully open blossom	31,700,976	30,410,918	9.51	9.12	92.93	42.42
SRR10076832 (leaf)	54,351,063	53,782,021	16.10	15.48	90.21	43.15

### RNA library construction and deep sequencing

The sample mixtures were lysed with 1 mL TRIzol reagent (Invitrogen, Carlsbad, CA, USA). Total RNA was prepared according to the manufacturer’s instructions. RNA purity was evaluated with a NanoPhotometer spectrophotometer (Implen USA, Westlake Village, CA, USA). The RNA concentrations were measured with a Qubit RNA assay kit and a Qubit 2.0 fluorometer (Life Technologies, Waltham, MA, USA). RNA integrity was evaluated with the RNA Nano 6000 assay kit in an Agilent Bioanalyzer 2100 system (Agilent Technologies, Santa Clara, CA, USA). RNA samples passing the quality control test were stored at − 20 °C until later use.

The RNA library was constructed by the Novogene Co. Ltd. (Beijing, China). Three micrograms RNA per sample was used as the input material for library preparation. Polyadenylated (poly(A)) RNA was purified from total RNA with poly T oligo-attached magnetic beads. Fragmentation was carried out using divalent cations under elevated temperature in NEBNext First Strand Synthesis Reaction Buffer (5X). After reverse transcription, the fragments were purified with AMPure XP system (Beckman Coulter, Beverly, USA), and the cDNA fragments with length in 150–200 bp were preferentially selected for PCR. At last, PCR products were purified (AMPure XP system) and library quality was assessed on the Agilent Bioanalyzer 2100 system. RNA sequencing was performed in an Illumina HiSeq 4000 system (Illumina, San Diego, CA, USA) using the 125-bp, strand-specific, paired-end mode.

### RNA-seq data preprocessing

Before proceeding to the transcriptome assembly, the RNA-seq raw data were filtered with Trimmomatic^10^ to remove adaptor-contained reads, N-content > 10% reads, and low-quality reads which the percent of base with sequence quality SQ ≤ 5 is more than 50% of the whole reads.

### Construction of the integrative transcriptome

The integrative transcriptome for *Bougainvillea* was constructed with TransIntegrator^11^ by integrating multiple RNA-seq transcriptomes. In this study, we defined a new term “integrative transcriptome” as the collection of all transcripts expressed in different tissues. It is kind of combination of transcriptomes but much more than that. The algorithm of TransIntegrator has been well described in^11^, and we only specified the parameters of current application briefly here. In principle, it took four continuous steps for integrative transcriptome construction as follows: (1) 20 Illumina RNA sequencing datasets were de novo assembled separately with Trinity software (version 1.0; Parameters: contig length > 200 bp, K-mer > 5, and max reads of per graph > 200 bp)^12^. (2) Subsequently, all expressed transcripts of the transcriptomes were mixed together and clustered with CD-HIT-EST (version 4.5.4)^13^ by setting a sequence identity threshold -c > 90% and an alignment coverage -aS > 80%. The clustered sequences were identified, the longest sequences were elected as the representatives, and the shorter sequences were discarded. (3) The representative transcripts were bridged with CAP3 (version 12/21/07, default parameters) to form longer sequences^14^ if two sequences had > 40 bp overlap (> 90% sequence identity). (4) The integrative transcriptome for *Bougainvillea* was refined by removing sequences < 300 bp.

The conventional procedure was adopted to annotate the integrative transcriptome: The coding transcripts were identified with Annocript (version 1.1.3; default parameters)^15^ by referring to the SwissProt and Pfam databases. The rRNA was annotated with RNAmmer (version 1.0.0; default parameters)^16^. The tRNA was annotated with tRNAscan-SE (version 2.1.3; default parameters)^17^. The ncRNAs and miRNA precursors were identified by using the BLAST tool (version 1.3.1; e-value: 1e−5, identify: 90%) against the NONCODE database^18^ and miRBase^19^. All annotations were integrated by discarding the low confident annotations subject to the default threshold of corresponding annotation tools.

### Validation of the integrative transcriptome

We validated the integrative transcriptome using both computational and experimental methods. Computationally, we mapped the transcriptomic reads against all assembled transcripts with BLASTn to confirm every bases of the transcripts were supported by multiple (≥ 5) reads. In addition, we assessed the integrative transcriptome (as the gene sets) with BUSCO (version 4.1.4; name = “embryophyta_OthoDB10”)^20^. Moreover, we selected a list of transcripts for experimental validation by meeting criteria of: (1) some transcripts are comparatively dissimilar (identify < 50%) to their matched *A. thaliana* homologs. (2) Some transcripts are significantly longer or shorter than their *A. thaliana* homologs. (3) And the transcript sequence can be determined by single read-through of Sanger sequencing. Accordingly, eight transcripts were selected for experimental validation, including three dissimilar transcripts (NBP35, RGL2, and ATZNMP) to the matched *A. thaliana* homologs, four shorter transcripts (PAF2, PGY2, NBP35, and ATZNMP), three longer transcripts (EXO70C1, IMPA, and RGL2), and one nearly identical transcript (PRT6). The selected eight transcripts were first reversely transcribed into cDNAs (TransGen, China) from the extracted total RNA samples and further amplified by the Polymerase Chain Reaction (PCR) with specifically designed primers (Supplementary Table 1). The PCR products were checked for band size on agarose gel. Furthermore, the sequences of right size PCR products were determined by the Sanger sequencing.

### The integrative transcriptome-based transcriptome assembly and performance evaluation

Usually, the reference-based transcriptome assembly exhibits more accurate and more stable performance than that of de novo approaches. In this study, to make the integrative transcriptome an alternative reference, it was annotated and preformatted as two files: BougainvilleaXbuttiana_Manila.fa and BougainvilleaXbuttiana_Manila.gtf. These two files can be downloaded from the project website. Subsequently, we carried out the reference-based transcriptome assembly using the processed integrative transcriptome as the alternative reference: reads aligned with HISAT2 (version 2.1.0; default parameters)^21^, assembled and estimated transcript abundances with StringTie (version 1.3.4d; default parameters)^22^, and quantified with R package Ballgown (default parameters)^23^.

The quality of transcriptomes assembled by different methods were evaluated by examining the completeness and fragmentation ratio of the transcripts with BUSCO (version 4.1.4; database = “embryophyta_OthoDB10”, number of BUSCOs = 1614)^20^. Before the BUSCO evaluation, we excluded transcripts < 300 bp from the transcriptomes under the consideration of significant difference in transcript length distribution (Fig. 4a). In addition, the potential bridge and assembly score were determined with TransRate (version 1.3, default parameters)^24^. A good quality transcriptome is expected to have larger transcript completeness, smaller fragmentation ratio, less potential bridges, and higher assembly score. As the controls, we also demonstrated de novo transcriptome assembly, the only applicable solution up-to-date, with Trinity software (version 2.8.5; default parameters) and Velvet (version 1.2.10; default parameters)^25^. The quality assessment was conducted on an external Illumina sequencing datasets, which was determined in young leaves of *Bougainvillea* × *buttiana* (SRA: SRR10076832), the only transcriptomic experiment of same species as that of this study. For fair evaluation, this external dataset was also incorporated into the integrative transcriptome to capture the leaves-specifically expressed transcripts.

### Database construction

For user convenience, the transcript library was presented as an online interactive database called InTransBo, which was constructed on the Linux–Apache–JSP platform. MySQL software was used to manage data storage, access, and maintenance. Efficient and friendly user interfaces were designed with JavaScript for interactive transcript search and retrieval.

### Statement of consent

This study on *Bougainvillea* complies with relevant institutional, national, international guidelines and legislation. All the samples in this study were collected from the pot-culture *Bougainvillea* provided by The Wanyin Environmental Technology Limited Company.

## Results and discussion

### Construction and validation of the integrative transcriptome

**Figure 1:**
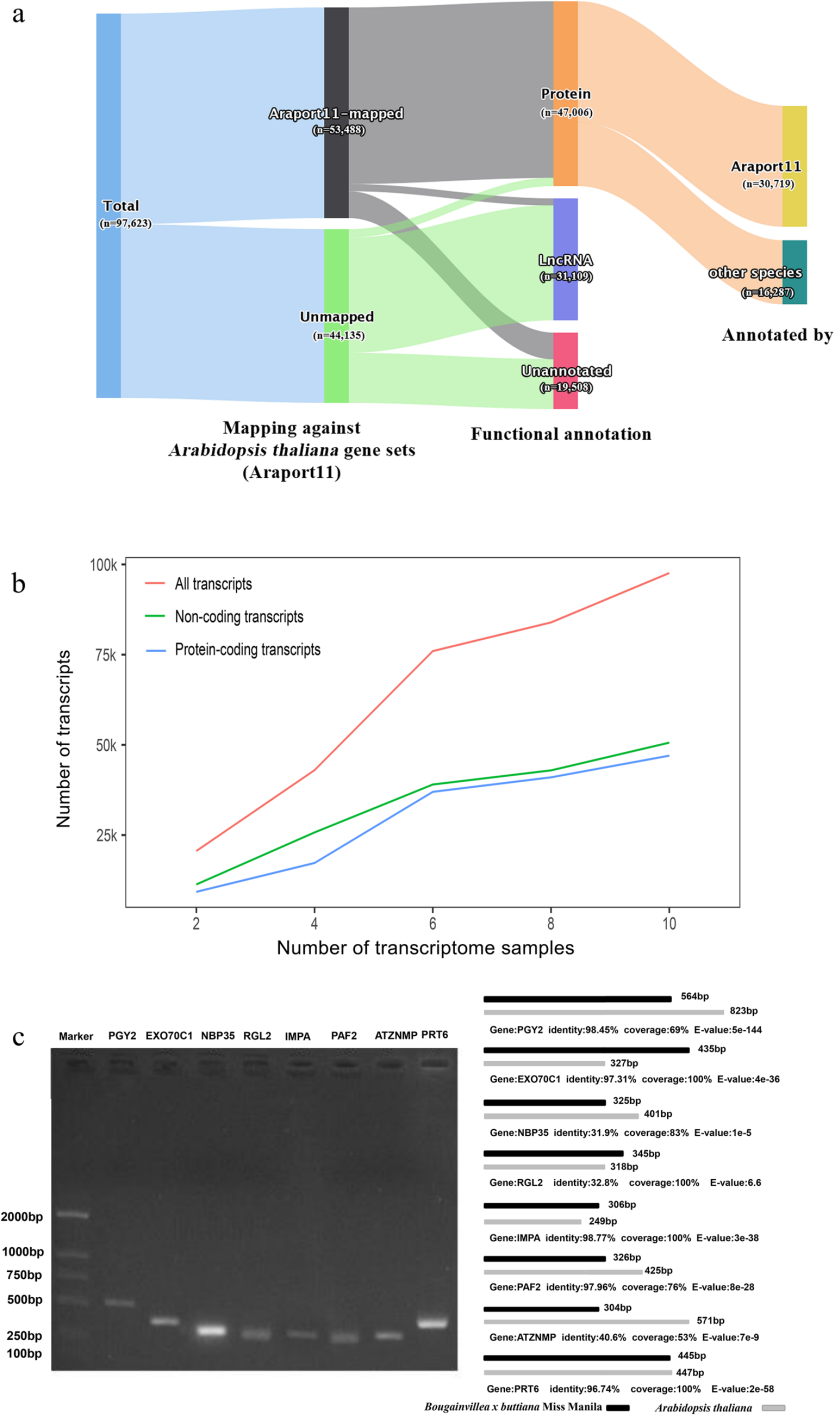
Overview of the integrative transcriptome. (**a**) Functional annotation of the integrative transcriptome. (**b**) The scalability of integrative transcriptome. (**c**) Experimental validation of selected eight transcripts using PCR and Sanger sequencing.

After quality control, the 20 Illumina sequencing transcriptome datasets generated ~ 590.30 million clean reads and 172.23 gigabase pairs (Gbp). Detailed information of these datasets was summarized in Table 1. Integration of these datasets with TransIntegrator yielded a collection of 97,623 non-redundant transcripts expressed in multiple tissues, which composed of the integrative transcriptome of *Bougainvillea* (Fig. 1). The average GC content of the integrative transcriptome was 40.12. The average sequence length was 724.11 bp. About 77.03% of the transcripts were 300–885 bp in length, which agreed with the previous estimation of plant gene length of 183–2000 bp^26^. Of the 97,623 sequences, ~ 48.15% (47,006) were annotated as protein-coding transcripts, ~ 31.87% (31,109) were putative long non-coding RNAs (lncRNAs) and other non-coding RNAs (ncRNAs), and the remaining 19.98% (19,508) were not annotated (Fig. 1a). Noteworthy, the integrative transcriptome can expand in size when involving more Illumina sequencing transcriptomes; the more diverse conditions, the more complete integrative transcriptome we can have. However, the protein-coding transcripts stopped growing rapidly after integration of six transcriptomes in this study (Fig. 1b).

To validate the integrative transcriptome, we conducted the reads mapping analysis. The analysis confirmed every single bases of transcripts were supported by multiple (≥ 5) mapping reads. We also performed a gene conservation analysis by mapping the transcripts of *Bougainvillea* integrative transcriptome against the gene sets (48,267 genes) of *Arabidopsis thaliana* genome (Araport11, release 2021–06-28) with BLASTx by the criteria of sequence identity > 30%. As the results, 53,488 *Bougainvillea* transcripts can be mapped to 39,555 (about 81.95%) gene sets of *A. thaliana*, including 44,855 protein-coding transcripts, 1827 putative lncRNAs, and 6808 unannotated transcripts. For the remaining 44,135 unmapped transcripts, 2390 were potential protein-coding, 29,282 were putative lncRNAs, and 12,702 were unannotated. The unmapped transcripts were either *Bougainvillea*-specific genes or wrongly assembled transcripts. In addition, of 47,006 protein-coding transcripts of *Bougainvillea*, 30,719 were annotated subject to *A. thaliana* genes, and the remaining 16,287 transcripts were more similar to homologous genes of other species (Fig. 1a).

Moreover, we selected eight transcripts for RT-PCR amplification and subsequent Sanger sequencing. These eight transcripts included three dissimilar transcripts (NBP35, RGL2, and ATZNMP) to the matched *A. thaliana* homologs, four shorter transcripts (PAF2, PGY2, NBP35, and ATZNMP), three longer transcripts (EXO70C1, IMPA, and RGL2), and one nearly identical transcript (PRT6). To this end, all transcripts were validated with the same size and same sequence as they were compiled (Fig. 1c). This result manifested the selected transcripts were well assembled. Putting all the results together, the integrative transcriptome was mostly reliable.

### Construction of the integrative transcriptome database for *Bougainvillea*

**Figure 2:**
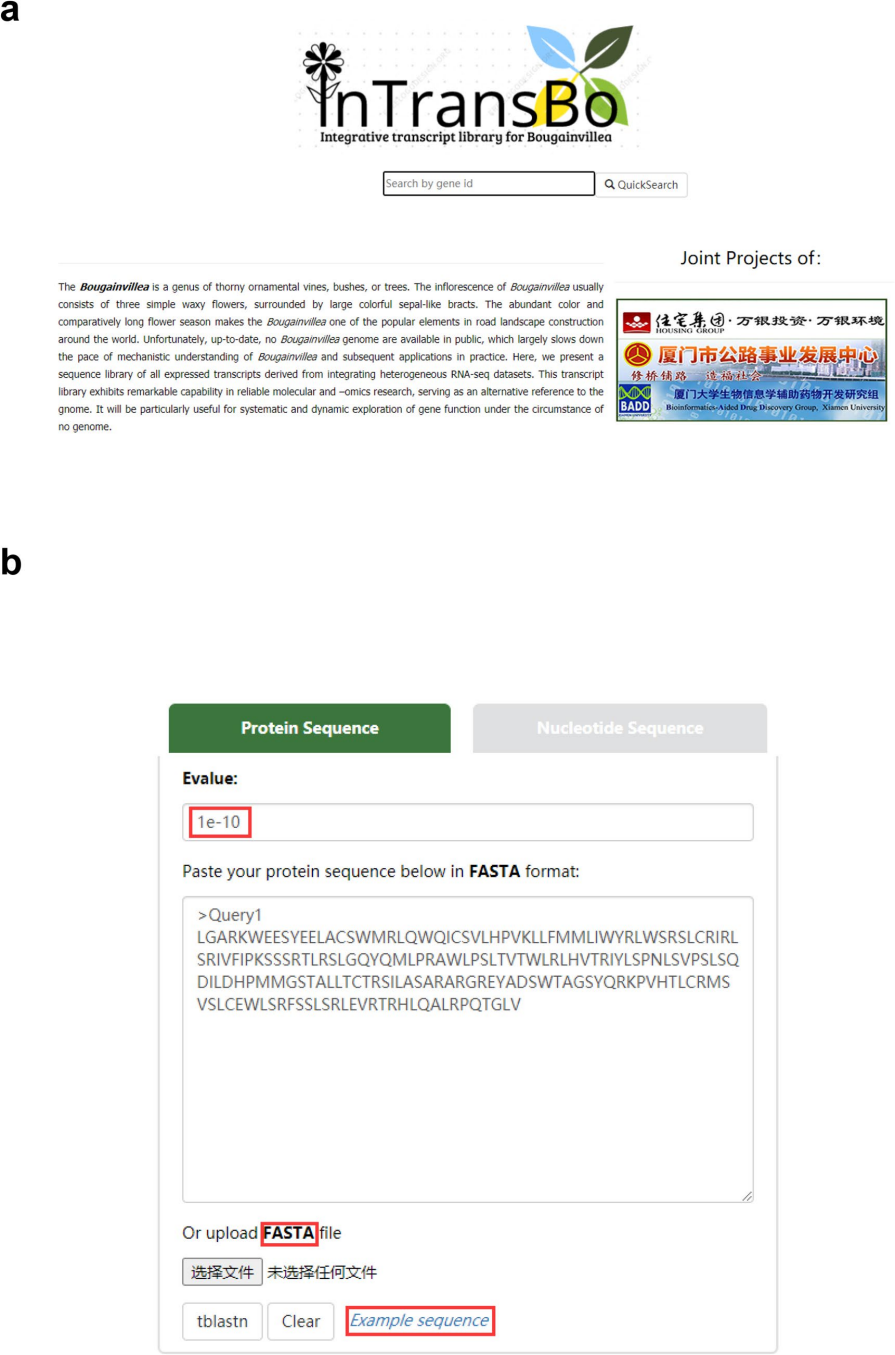
Snapshots of InTransBo user interfaces. (**a**) Homepage and keyword search form. (**b**) BLAST search form.

**Figure 3:**
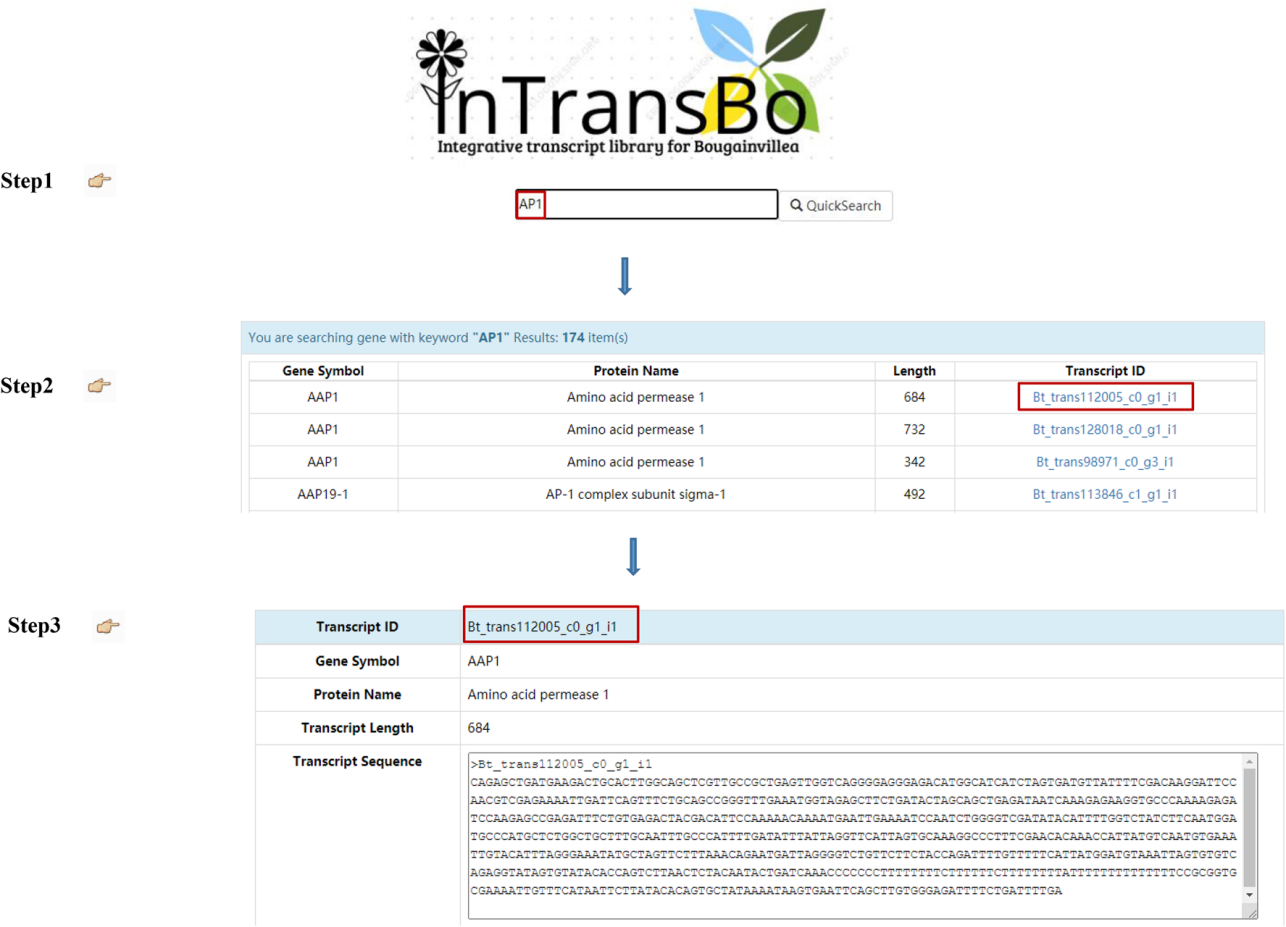
Data retrieval by keyword search method.

In this study, we constructed the integrative transcriptome database for *Bougainvillea* (*Bougainvillea* × *buttiana* Miss Manila), namely InTransBo. The InTransBo database is accessible freely at http://www.bio-add.org/InTransBo/ or at its mirror site http://bioinf.xmu.edu.cn/InTransBo/. InTransBo uses keyword search and sequence BLAST to retrieve data interactively. The keyword search function allows both accurate and fuzzy transcript search via the input of complete or partial gene symbols, gene names, protein names, and abbreviated protein names (Fig. 2a). The search engine returns the hit terms in alphabet order along with the gene symbol, protein name, transcript length, and transcript ID. Clicking on the transcript ID redirects to the transcript information page containing various data listed in order including transcript annotation, sequence information, and transcript expression profiles over different tissues (Fig. 3).

For newly identified sequences and unnamed sequences, InTransBo enables data access via an alternative BLAST method. The database supports BLASTn and tBLASTn to identify nucleic acid and protein sequences, respectively. The input may either be the typing sequence in text form or an uploaded file in FASTA format (Fig. 2b). The embedded BLAST engine responds to all hit sequences meeting the default expectation threshold of E-value = 1e−10 and is sorted by hit score (Fig. 2b). For each hit, alignment details may be obtained using the “Alignment” hyperlink. The detailed information of hits may be acquired via the transcript ID hyperlinks. The integrative transcriptome is free for downloading; however, user registration is required. The integrative transcriptome was also annotated and preformatted as a gff file with Annoscript to make it an alternative reference for omics research.

### The potential application of integrative transcriptome in transcriptome assembly

**Figure 4:**
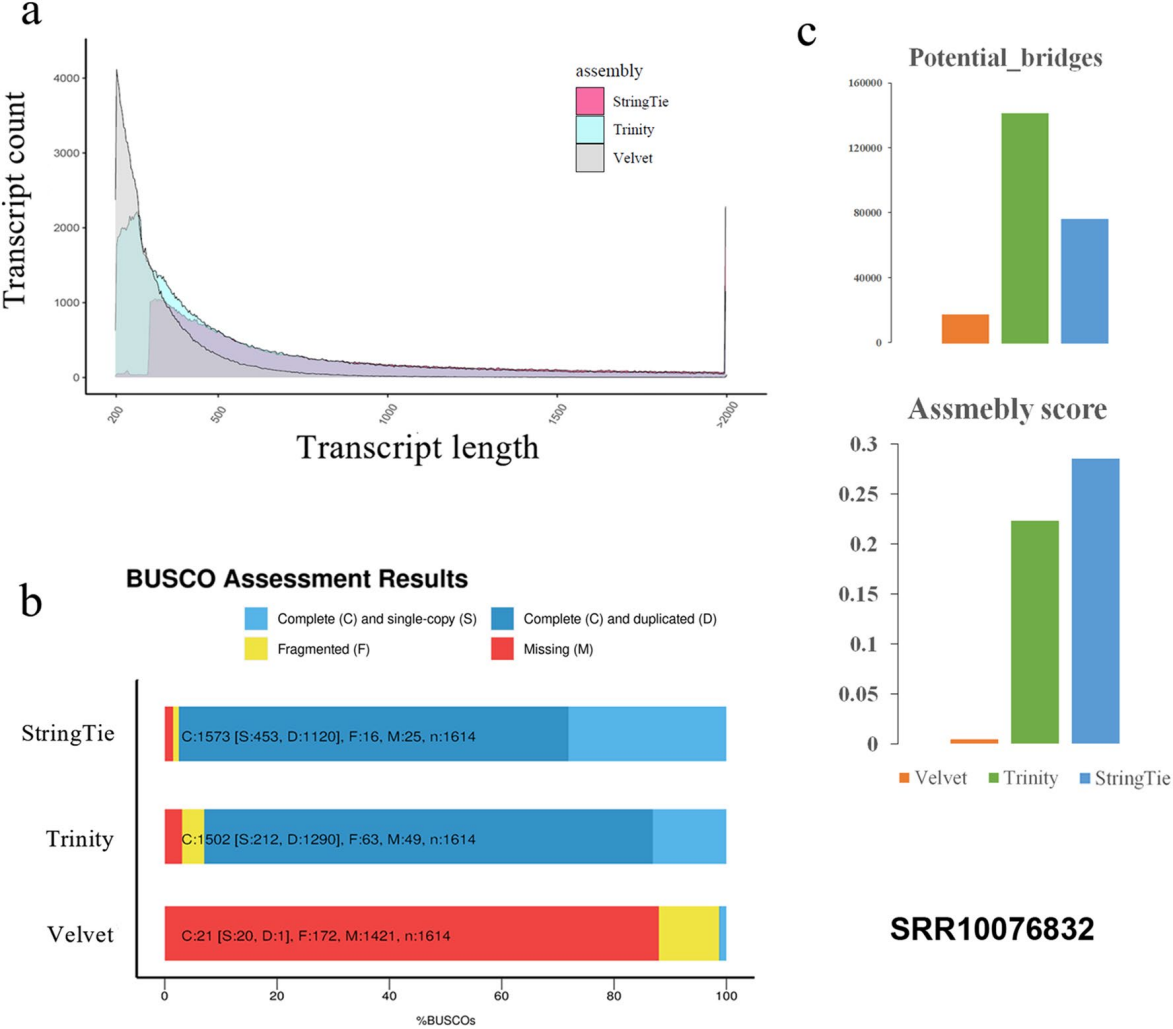
Quality comparison of transcriptomes assembled with the integrative transcriptome-referenced method (using the StringTie assembler) and with de novo assemblers (Trinity and Velvet). An external Illumina sequencing dataset of *Bougainvillea* × *buttiana* (SRA: SRR10076832) was involved in the comparison. The quality of transcriptome was evaluated with BUSCO and TransRate. (**a**) The transcript length distribution of transcriptomes assembled by three methods. (**b**) BUSCO evaluation of transcriptomes. (**c**) TransRate evaluation of transcriptomes.

As a potential application, we applied the integrative transcriptome to reference-based transcriptome assembly with the HISAT2 + StringTie pipeline. The assembled transcriptomes were further compared with those assembled by the de novo assemblers (Trinity and Velvet). The comparison was conducted on an external Illumina sequencing datasets determined in same species of this study (SRA: SRR10076832) (Table 1). The transcriptome quality was mainly evaluated by BUSCO and TransRate. As shown in Fig. 4, both de novo methods produced much more transcripts than that of the StringTie method; however, majority of the transcripts assembled by Trinity and Velvet were < 300 bp and consisted of a large number of duplicates. The BUSCO evaluation manifested that the StringTie method outperformed Trinity and Velvet by owning more complete transcripts and less duplicates (Fig. 4b). In this case, Velvet failed to produce reliable transcriptome from almost all aspects. Similar to the evaluation of BUSCO, additional TransRate evaluation estimated higher assembly score and less potential bridges in StringTie-assembled transcriptome. Therefore, the integrative transcriptome can serve as a good reference in transcriptome assembly under the circumstance of no genome.

Taking the integrative transcriptome as the reference, we portrayed the gene expression landscapes for ten *Bougainvillea* flower tissues, which can be acquired in the InTransBo database via specifying gene symbols. These gene expression profiles are valuable for exploring the biological basis of *Bougainvillea*. For instance, flower is one of the major sources of metabolites, natural dyes, pigments, medicinal uses in *Bougainvillea*. Via comparing to 27 genes related to the meristem response and development in *Arabidopsis thaliana*^27^, we identified 13 distinct homologous genes (52 transcripts) in *Bougainvillea*. They were AGL24, AP1, CAL, FUL, LFY, SOC1, SPL3, SPL4, SPL5, SPL9, SPL15, FD, and SVP. Monitoring the behaviors of these genes in different flower tissues will provide insights into the molecular mechanisms during floral transition of *Bougainvillea*.

## Conclusion

*Bougainvillea* sp. require systematic transcriptome research but lack genome support. Regretfully, whole-genome sequencing is not a viable option to-date as it is costly and technically impracticable. Here, we used the computational method TransIntegrator to construct an integrative transcriptome, the collection of all expressed transcripts, for *Bougainvillea* by integrating 20 heterogeneous Illumina sequencing datasets. Both computational and experimental methods validated that the integrative transcriptome was well compiled. Based on the integrative transcriptome, we curated the InTransBo database for interactive data retrieval. To the best of our knowledge, the InTransBo database could be the first comprehensive genomic and transcriptional resource for *Bougainvillea*. Furthermore, we demonstrated a typical application of the integrative transcriptome as an alternative reference in transcriptome assembly, which significantly outperformed the de novo methods, the only applicable solution up-to-date under the circumstance of lacking genome.

It is also acknowledged that the integrative transcriptome has its limitations and is far away from completeness. There may exist some falsely assembled long transcripts due to wrongly bridging fragmented transcripts from different transcriptomes, even though the bridges may be supported by multiple reads. Moreover, the de novo assembly may account for a portion of false transcripts, which can’t be properly solved by the TransIntegrator. It is thus desired that long-read sequencing datasets could be introduced and more sequencing datasets of different spatiotemporal samples could be involved to largely improve the quality and completeness of integrative transcriptome.

Nevertheless, the integrative transcriptome could mitigate the genome constraint on systematic investigation of *Bougainvillea* by serving as a reliable surrogate to the genome. It will help elucidate the molecular basis of *Bougainvillea* and facilitate precise flower regulation in horticultural practices. The same strategy could be readily applied toward the systematic exploration of other plant species lacking adequate genomic data.

## Supplementary Information

Supplementary Table S1.

## Data Availability

The Illumina sequencing datasets determined in this study have been deposited in the NCBI SRA under the accession number PRJNA747624 and PRJNA719248. The data are publicly accessible at https://www.ncbi.nlm.nih.gov/sra.
