# Supplementary material for: Construction of integrative transcriptome to boost systematic exploration of Bougainvillea
Source: Sci Rep. 2022 Jan 18;12:923. doi: 10.1038/s41598-022-04984-8 (PMC8766500; doi:10.1038/s41598-022-04984-8)
Supplement: Supplementary file 1 — Supplementary Table S1. [file 41598_2022_4984_MOESM1_ESM.docx]

| Primer_ID | *Primer(5'--3') | gene |
| --- | --- | --- |
| >Bt_trans813_c0_g1_i1-F | CCCTCATCGACAACCCATCTC | PGY2 |
| >Bt_trans813_c0_g1_i1-R | CCTTGTTTCGCACCCGGCAG |  |
| >Bt_trans7901_c0_g1_i1-NF | CCTCCTAGCCATGGACATATTC | EXO70C1 |
| >Bt_trans7901_c0_g1_i1-NR | GGGTTCAACAGAGATCCATC |  |
| >Bt_trans64870_c0_g1_i1-F | CTCGACCAACGCCAACCTG | NBP35 |
| >Bt_trans64870_c0_g1_i1-R | GGGGGATACCCTCAGTAATG |  |
| >Bt_trans73247_c0_g1_i1-F | CCGCCATCATGCTCAAGAAC | RGL2 |
| >Bt_trans73247_c0_g1_i1-R | TGCCAGTGTCGGTAGCCTTG |  |
| >Bt_trans89440_c1_g1_i1-F | GGCAAAGAAAGGAGAGAGGC | IMPA-9 |
| >Bt_trans89440_c1_g1_i1-R | GCACTGCACAAGAATTGGC |  |
| >Bt_trans91237_c1_g1_i1-F | CGCATGTAGCGGGAGAGAAC | PAF2 |
| >Bt_trans91237_c1_g1_i1-R | CAAAGCCCCATTTTCCTCTC |  |
| >Bt_trans93128_c0_g1_i1-F | CCCACTCAAGTTAACTATG | ATZNMP |
| >Bt_trans93128_c0_g1_i1-R | CCCAATGATTGCTTTAGCAAG |  |
| >Bt_trans94188_c1_g1_i1-F | CAAAGACAACAGACATGTG | PRT6 |
| >Bt_trans94188_c1_g1_i1-R | CGGGAATGCTTAAGTATTC |  |

**Table S1**. The primers of selected eight transcripts used for PCR.
